# Intracellular expression of IRF9 Stat fusion protein overcomes the defective Jak-Stat signaling and inhibits HCV RNA replication

**DOI:** 10.1186/1743-422X-7-265

**Published:** 2010-10-12

**Authors:** Bret Poat, Sidhartha Hazari, Partha K Chandra, Feyza Gunduz, Xavier Alvarez, Luis A Balart, Robert F Garry, Srikanta Dash

**Affiliations:** 1 Department of Pathology and Laboratory Medicine, Tulane University Health Sciences Center, 1430 Tulane Avenue, New Orleans, LA-70112, USA; 2 Section of Gastroenterology and Hepatology, Tulane University Health Sciences Center, 1430 Tulane Avenue, New Orleans, LA-70112, USA; 3 Department of Microbiology and Immunology, Tulane University Health Sciences Center, 1430 Tulane Avenue, New Orleans, LA-70112, USA; 4 Division of Comparative Pathology, Tulane National Primate Research Center, 18703 Three Rivers Road, Covington, LA-70433, USA

## Abstract

Interferon alpha (IFN-α) binds to a cell surface receptor that activates the Jak-Stat signaling pathway. A critical component of this pathway is the translocation of interferon stimulated gene factor 3 (a complex of three proteins Stat1, Stat2 and IRF9) to the nucleus to activate antiviral genes. A stable sub-genomic replicon cell line resistant to IFN-α was developed in which the nuclear translocation of Stat1 and Stat2 proteins was prevented due to the lack of phosphorylation; whereas the nuclear translocation of IRF9 protein was not affected. In this study, we sought to overcome defective Jak-Stat signaling and to induce an antiviral state in the IFN-α resistant replicon cell line by developing a chimera IRF9 protein fused with the trans activating domain (TAD) of either a Stat1 (IRF9-S1C) or Stat2 (IRF9-S2C) protein. We show here that intracellular expression of fusion proteins using the plasmid constructs of either IRF9-S1C or IRF9-S2C, in the IFN-α resistant cells, resulted in an increase in Interferon Stimulated Response Element (ISRE) luciferase promoter activity and significantly induced HLA-1 surface expression. Moreover, we show that transient transfection of IRF9-S1C or IRF9-S2C plasmid constructs into IFN-α resistant replicon cells containing sub-genomic HCV1b and HCV2a viruses resulted in an inhibition of viral replication and viral protein expression independent of IFN-α treatment. The results of this study indicate that the recombinant fusion proteins of IRF9-S1C, IRF9-S2C alone, or in combination, have potent antiviral properties against the HCV in an IFN-α resistant cell line with a defective Jak-Stat signaling.

## Introduction

Hepatitis C virus (HCV) infection is a major public health problem with 170 million infected individuals worldwide [1,2]. HCV infection establishes a chronic inflammatory liver disease in over 70 percent of patients. In chronically infected individuals the disease slowly progresses over decades resulting in liver cirrhosis, hepatocellular carcinoma (HCC), and liver failure [3]. The World Health Organization estimates that 3% of the world's population is infected with HCV and approximately three to four million new cases of HCV infection occur globally per year [4]. Pegylated IFN-α plus ribavirin is the standard of care for the treatment of chronic HCV infection [5,6]. Approximately one half of treated patients clear the virus infection with this regimen and as many as 20% of patients prematurely discontinue therapy due to side effects [7]. The molecular details that determine response to treatment are not well understood.

IFN-α signal transduction is initiated by the binding of the IFN-α molecule to its surface receptor. This binding activates the receptor associated tyrosine kinases, Janus kinase 1 (Jak-1) and tyrosine kinase 2 (Tyk2), which then phosphorylate Stat1 and Stat2 proteins. Phosphorylated Stat1 and Stat2 then disassociate from the receptor and form the hetero-trimeric IFN-stimulated gene factor 3 (ISGF3) complex which then translocates into the nucleus and induces antiviral gene transcription. This cascade of biochemical reactions occurring in normal cells following IFN-α treatment is called the Jak-Stat pathway [8].

The mechanism of IFN-α resistance has been described to be related to several viral and host related factors [9-11]. For this purpose, we have developed multiple IFN-α resistant cell lines containing sub-genomic HCV RNA as a model to study IFN resistance. Characterization of these cell lines have revealed that Jak-Stat pathway defects give rise to the IFN-α resistant phenotype [12,13]. A more recent analysis revealed that each of our nine resistant cell lines express a truncated IFN-α receptor 1 (IFNAR1), resulting in the functional inactivation of the IFN-α receptor (unpublished data). A fusion product of IRF9 to the TAD of either Stat1 or Stat2 was previously engineered [14]. We show here that intracellular expression of an IRF9-Stat fusion protein in an IFN-resistant replicon cell line bypasses cellular defects and induces transcription of the genes under the control of the interferon sensitive response element (ISRE) promoter. Furthermore, we show here that intracellular expression of these constructs in an IFN-α resistant cell line containing sub-genomic HCV RNA inhibited viral replication and viral protein expression and induced HLA-1 surface expression without IFN-α treatment. These studies provide evidence that targeting the Stat1 or Stat2 proteins can induce an intracellular antiviral state independent of the IFN treatment thus providing an alternative strategy to overcome HCV resistance to standard IFN-α based therapy.

## Materials and methods

### IFN-α resistant HCV replicon cell lines

IFN-α resistant cells containing sub-genomic HCV genotype 1b RNA were generated in our laboratory by prolonged treatment of the low inducer replicon cell line Con-15-3 with IFN-α as described previously [12]. IFN sensitive S9-13 cells containing sub-genomic HCV genotype 1b HCV RNA were kindly provided by Ralf Bartenschlager, University of Heidelberg, Heidelberg, Germany. Stable cell lines, S3-GFP (IFN sensitive) and R4-GFP (IFN resistant) containing HCV genotype 2a subgenomic RNA with a green fluorescence protein (GFP) fusion in frame with HCV NS5A were prepared in our laboratory as previously described [13]. All cell lines were maintained in Dulbecco's modified Eagle's medium (DMEM) supplemented with 2 mM L-glutamine, nonessential amino acids, 100 U/ml of penicillin, 100 μg/ml of streptomycin, 500 μg/ml of G418, and 5% fetal bovine serum.

### Plasmid constructs and DNA transfection

**Figure 1:**
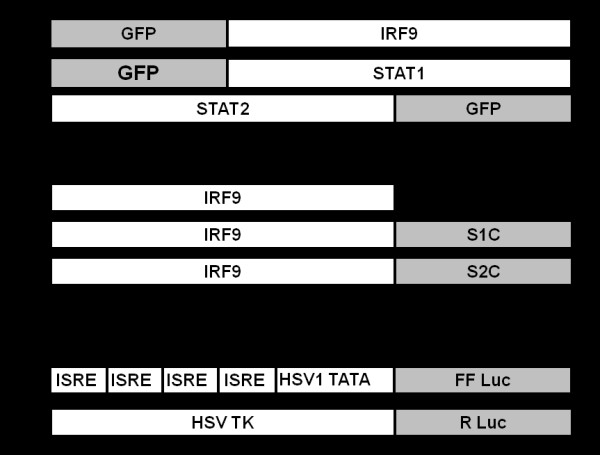
**Structure of plasmid constructs**. **(A) **The plasmid constructs containing the full-length clones of IRF9, Stat1 and Stat2 fused with GFP either at the N-terminal or C-terminal ends. **(B) **Full length IRF9 clone fused with the TAD of either Stat1 or Stat2. IRF9-S1C contains the full length IRF9 molecule fused in frame with the 38 C-terminal amino acids of Stat1. IRF9-S2C also contains the full length IRF9 molecule fused in frame with the 104 C-terminal amino acids of Stat2. (**C) **The ISRE reporter plasmid containing four copies of ISRE sequences positioned upstream of the HSV thymidine kinase (TK) promoter TATA box that drives the expression of FL. The bottom construct, used as a transfection control contains RL downstream of a HSV TK promoter.

Three different expression plasmid constructs (Fig. 1) were used in this study and assessed for their ability to overcome defective Jak-Stat signaling and to induce an antiviral state in R15-3 cells. The first plasmid, pcDNA3-IRF9 contains the open reading frame of the human IRF9 gene under the control of a cytomegalovirus (CMV) promoter. The second plasmid, IRF9-S1C contains the 38 C-terminal amino acids of Stat1 protein fused in frame with the human IRF9. The third plasmid construct, IRF9-S2C contains the 104 C-terminal amino acids of Stat2 fused in frame with the human IRF9 coding sequence. All three recombinant plasmid constructs were obtained as a gift from Curt Horvath, Feinberg School of Medicine, Northwestern University, Evanston, Illinois [14]. The ISRE-Luciferase plasmid was obtained from Steve Goodbourn, St.George's Hospital, University of London, London, UK [15]. The pRL-TK plasmid was obtained from Promega Corporation, Madison, WI. The Stat1-GFP and IRF9-GFP constructs were obtained from Addgene Inc, Cambridge, MA [16]. The Stat2-GFP construct was obtained from Dr. Mario Koster Braunschweig, Germany [17]. DNA transfections were performed using Fugene-6 transfection reagent following the manufacturer's recommendations (Roche Molecular Diagnostics, Indianapolis, IN).

### Luciferase Reporter Assays

The R15-3 cell line was transfected using either IRF9, IRF9-S1C, or IRF9-S1C plus IRF9-S2C plasmid, ISRE firefly-luciferase (FL) plasmid, pRL-TK renilla-luciferase (RL) plasmid and treated with or without IFN-α (Schering, Kenilworth, NJ). At 24 hours post-transfection FL and RL activity was measured by integrating the total light emission over ten seconds with a luminometer (Luman LB9507, EG&G Bethold, Berlin, Germany). The results were normalized by dividing the FL value with the RL value for the same sample.

### Nuclear Translocation Assay

R15-3 and 9-13 cells were were plated in a two well Lab-Tek chamber slide (Electron Microcopy Sciences, Hatfield, PA) at a density of 5 × 10^4 ^cells per ml. Twenty four hours later, cells were transfected with 1 μg of the respective Stat1-GFP, Stat2-GFP or IRF9-GFP plasmid DNA using Fugene-6 transfection reagent. At 48 hours post- transfection, To-Pro3 nuclear marker (Invitrogen, Molecular Probes, Oregon) was added to the samples at 1 μg/ml, and incubated for five minutes in PBS. IFN-α (1000 IU/ml) was then added to the appropriate groups. Confocal microscopy was performed using a Leica TCS SP2 confocal microscope equipped with three lasers (Leica Microsystems, Exton, PA). Optical slices were collected at 512 × 512 pixel resolution. NIH Image version 1.62 and Adobe Photoshop version 7.0 were used to assign correct colors of channels collected, including the Green Fluorescent Protein (green), To-Pro3 633 (far red), and the differential interference contrast image (DIC) (gray scale).

### HLA-1 Expression by Flow Cytometry

R15-3 and 9-13 cells were transfected with IRF9, IRF9-S1C, IRF9-S2C, or IRF9-S1C plus IRF9-S2C plasmid. At 48 hours post-transfection the cultured cells were suspended in 100 μl of PBS and 20 μl of phycoerythrin conjugated mouse anti-human HLA-ABC antibody (Pharmingen, San Jose, CA) and incubated for 15 minutes at 4°C. The cells were then resuspended in 500 μl of PBS and analyzed by a BD LSR-II flow cytometer (Becton-Dickinson, Franklin Lakes, New Jersey) using BD FACS Diva software.

### Ribonuclease Protection Assay (RPA) of the HCV Negative Sense Strand RNA

The antiviral properties of each IRF9 construct was evaluated by RPA detection of the HCV RNA negative sense strand utilizing a previously described method [15]. R15-3 cells were transfected with IRF9, IRF9-S1C, IRF9-S2C, or IRF9-S1C plus IRF9-S2C plasmid in the presence or absence of IFN-α (1,000 IU/ml). At 72 hours total RNA was isolated, hybridized, and analyzed by RPA.

#### Real-time RT-PCR

R15-3 cells were transfected with IRF9, IRF9-S1C, IRF9-S2C, or both IRF9-S1C, and IRF9-S2C plasmid. At 72 hours real time RT-PCR was used to quantify HCV RNA levels using a previously described method [13]. S9-13 and R15-3 cells with and without IFN-α (1,000 IU/ml) treatment were used as positive and negative controls respectively. Values represent the mean log copies of HCV RNA per μg of cellular RNA from three experiments. Error bars represent standard deviation (SD). The student's t-test was used to compare R15-3 transfected cells to R15-3 cells plus IFN-α (1,000 IU/ml). Values < 0.05 were considered significant.

### Immunocytochemical staining

R15-3 cells were transfected with IRF9, IRF9-S1C, IRF9-S2C or both IRF9-S1C, and IRF9-S2C, and were treated with or without IFN-α (1,000 IU/ml). At 48 hours the cells were mounted onto a glass slide and stained for HCV NS3 utilizing a previously described method [12].

### Analysis of Stat1 Phosphorylation by Co-immunoprecipitation

R15-3 and S9-13 cells were transfected with each of the plasmids listed in Fig. 1A. At 72 hours the cells were treated with or without IFN-α (1000 IU/ml). Forty-five minutes after IFN-α addition the cells were lysed by the addition of RIPA buffer with proteinase and phosphatase inhibitors (1 × PBS, 1% NP-40, 0.5% Deoxycholate, 0.1% SDS, 50 μg/ml PMSF, 5 μg/ml aprotinin, 5 μg/ml leupeptin, 1 μg/ml pepstatin). The GFP primary antibody (Santa Cruz Biotechnology, Santa Cruz, CA) was added to the lysate and rotated at 4°C overnight. The next morning protein A/G plus-agarose (Santa Cruz Biotechnology, Santa Cruz, CA) was added to each sample and rotated at 4°C for three hours. Western blotting was then performed using a Phospho-Stat1 Y701 (p-Stat1) primary antibody (Cell Signaling Technology, Danvers, MA) diluted (1:1,000) in blocking reagent, (0.1% tween 20, Tris buffered saline, and 5% NFDM) as described below.

### Immunoblot Blot Analysis

Cell lysates were prepared from IRF9, IRF9-S1C, IRF9-S2C, or both IRF9-S1C, and IRF9-S2C transfected R15-3 cells at 72 hours post-transfection. Western blot analysis was then performed using HCV NS5B (Abcam, Cambridge, MA), p-eIF-2α (Cell Signalling Technologies, Danvers, MA) and β-actin (Cell Signalling Technologies, Danvers, MA) primary antibodies.

### MTT Assay

The viability of IRF9 fusion construct transfected R15-3 cells was evaluated by the MTT assay. At 72 hours post-transfection 100 μl of the MTT solution (Sigma, St. Louis, MO) was added to the media in each well. After three hours of incubation at 37°C the media was aspirated and 1 ml of solubilization buffer (0.1 N HCL in absolute isopropanol) was added to each well. The absorbance of each sample was then measured at 570 nm utilizing a Beckman DU Spectrophotometer after blanking. The viability was then calculated by the formula dividing the OD_570 _of each sample by the mean OD_570 _of the controls.

## Results

### IFN-α independent nuclear localization of IRF9 in the R15-3 and S9-13 cells

**Figure 2:**
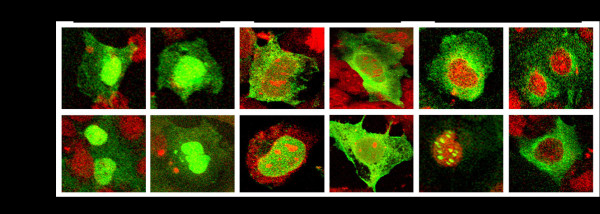
**Nuclear translocation of STAT-GFP constructs**. The S9-13 and R15-3 cell lines were transfected with IRF9, Stat1, or Stat2 GFP fusion constructs (-) and (+) IFN-α (1,000 IU/ml) and their nuclear translocation was observed under a confocal microscope. The images are represented as the superimposition of Green Fluorescent Protein (green), To-Pro3 633 (far red), and the differential interference contrast images (DIC) (gray scale). Fluorescence green and red microscopic picture of the same area were taken and superimposed using Abode Photoshop. **Left panel **shows high resolution picture showing that the IRF9-GFP fusion protein translocates to the nucleus of both S9-13 and R15-3 cells in an IFN-α independent manner. **Middle panel shows s**tat1-GFP fusion protein efficiently localized to the nucleus of S9-13 cells within 30 minutes after IFN-α treatment. Stat1-GFP was unable to localize to the nucleus in R15-3 cells. **Right panel **shows stat2-GFP efficiently localized to the nucleus of S9-13 cells within 30 minutes after IFN-α treatment. Stat2-GFP was unable to localize to the nucleus in R15-3 cells.

**Figure 3:**
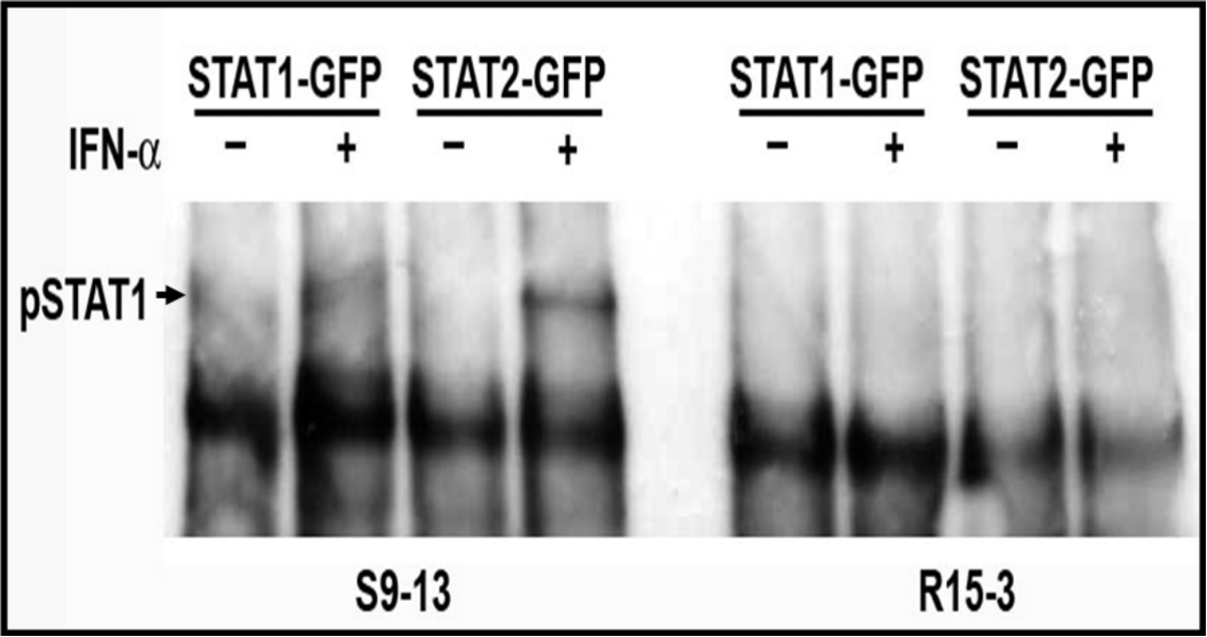
**Phosphorylation of Stat1-GFP and Stat2-GFP fusion proteins in S9-13 and R15-3 cells determined by co-immunoprecipitation**. S9-13 and R15-3 cells were transfected with either Stat1-GFP or Stat2-GFP. After 48 hours IFN-α (1,000 IU/ml) was added. Protein lysates were prepared, subjected to immunoprecipitation with a GFP antibody, and western blot analysis was performed using a p-STAT1 antibody.

The GFP fused Stat1, Stat2 and IRF9 plasmid clones illustrated in Fig. 1A were used to establish the dynamics of nuclear translocation in the S9-13 and R15-3 cells in the presence and absence of IFN-α. Using a transient transfection experiment, we demonstrate that both the Stat1-GFP and Stat2-GFP fusion proteins were expressed at high levels in the cytoplasm of both cell lines. Both the Stat1-GFP and Stat2-GFP fusion protein translocated to the nucleus of S9-13 cells 30 minutes after IFN-α treatment and returned to the cytoplasm by 24 hours (Fig. 2). However, the Stat1-GFP and Stat2-GFP proteins did not localize to the nucleus of the R15-3 cell line following IFN-α treatment. These results suggest that the nuclear translocation of the Stat1 and Stat2 proteins were impaired in the R15-3 cells. The IRF9-GFP fusion protein was expressed at intermediate levels in the S9-13 and R15-3 cell lines. The distribution of IRF9-GFP chimera protein was localized in both the cytoplasm and nucleus at all time points in both the R15-3 and S9-13 cell lines. These results suggest that the IRF9 protein diffuses freely from cytoplasm to the nucleus of both cell lines independent of IFN-α treatment. To understand the reason for differences in the nuclear translocation of Stat1 and Stat2-GFP fusion proteins in R15-3 and S9-13 cells, their phosphorylation status was examined after transfection. The co-immunoprecipitation experiments show that the Stat1-GFP and Stat2-GFP proteins were phosphorylated in the S9-13 cells but not in the R15-3 cells (Fig. 3).

### Intracellular expression of IRF9-Stat fusion protein activates ISRE transcription

**Figure 4:**
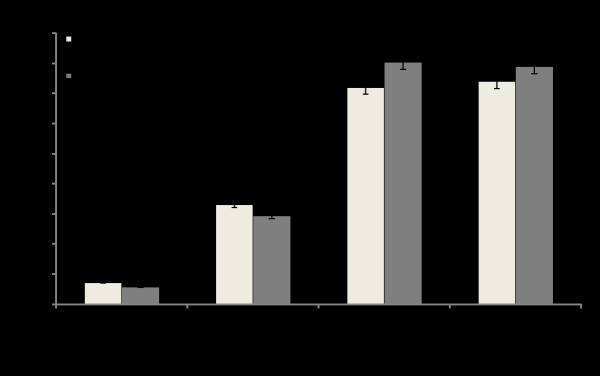
**Analysis of ISRE-luciferase promoter activation due to intracellular expression of IRF9-Stat fusion proteins**. R15-3 cells were transfected with IRF9, IRF9-S1C or IRF9-S2C plasmid and cultured (-) and (+) IFN-α (1,000 IU/ml). At 48 hours FL and RL activity were measured. Values are expressed in RL normalized units and error bars represent the SEM from three experiments. The Student's t-test was used to compare IRF9 with and without IFN to IRF9-S1C, IRF9-S2C and IRF9-S1C plus IRF9-S2C with and without IFN. Values <.05 were considered significant.

**Figure 5:**
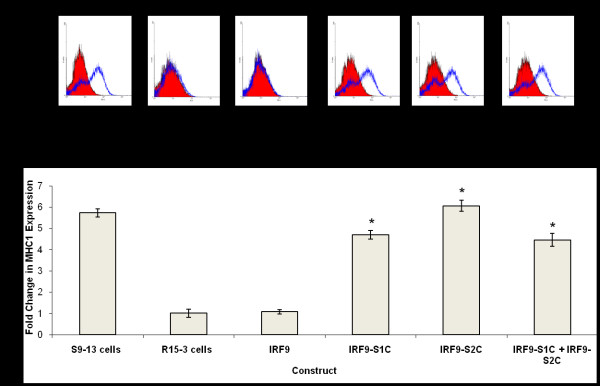
**Intracellular expression of IRF9-S1 or IRF9-S2C induced HLA-1 surface expression in the R15-3 cells**. S9-13 and R15-3 cells were transfected and cultured (-) and (+) IFN-α (1,000 IU/ml). After 48 hours, HLA-1 surface expression was quantified by flow cytometry. **(A) **Shows the HLA-1 surface expression in the IRF9-Stat fusion transfected R15-3 cells. The red cell population represents IFN-α naïve cells or untransfected cells and the blue cell population represents IFN-α treated or transfected cells. IRF9-S1C, IRF9-S2C, and IRF9-S1C plus IRF9-S2C transfected R15-3 cells demonstrated significant increases in HLA-1 surface expression. **(B) **Each value represents the mean fluorescence intensity from six experiments. The student's t-test was used to compare the fold increase of transfected R15-3 cells to untransfected R15-3 cells plus IFN-α. Asterisk (*) indicates transfected R15-3 cells with significantly different HLA-1 surface expression relative to untransfected R15-3 cells. Significance was considered at p-values < 0.05.

Since the IRF9-GFP construct efficiently localized to the nucleus of the R15-3 cells, we wanted to test whether fusing the TAD of either the Stat1 or Stat2 protein could induce IFN promoter activation. For this reason, expression plasmid constructs of IRF9 fused with the TAD of either Stat1 or Stat2 (Fig. 1B) were tested for their ability to activate the ISRE promoter in the R15-3 cell line. Intracellular expression of IRF9 after plasmid DNA transfection did not activate the ISRE-luciferase activity in the R15-3 cell line even after IFN-α treatment (Fig. 4), whereas the IRF9-S1C and IRF9-S2C fusion constructs significantly induced the ISRE-luciferase promoter (p < .025). The combination treatment with both IRF9-S1C and IRF9-S2C also showed high ISRE-luciferase activity in the R15-3 cells. There were no significant differences (p < .05 student's t-test) in ISRE-luciferase activity between the IFN-α (+) and (-) groups suggesting that the ISRE-luciferase induction was IFN independent. These results suggest that the fusion of the TAD of either Stat1 or Stat2 to IRF9 efficiently localized to the nucleus and activated the ISRE promoter in the R15-3 cell line. The results of the ISRE promoter induction experiments were confirmed by examining the HLA-ABC (HLA-1) surface expression of IRF9-Stat fusion transfected R15-3 cells by flow cytometry at 72 hours post-transfection. HLA-1 is an important molecule induced by the ISRE promoter which is involved in the immune recognition of virally infected cells [8]. Intracellular expression of either IRF9-S1C or IRF9-S2C induced HLA-1 surface expression in the R15-3 cells (Fig. 5A). The change in surface HLA-1 expression in R15-3 cells following plasmid transfection was quantified and compared to the fold change in HLA-1 surface expression of untransfected R15-3 cells after the addition of IFN-α (Fig. 5B). Significant increases (p < .05) in HLA-1 expression were observed for IRF9-S1C, IRF9-S2C and IRF9-S1C plus IRF9-S2C transfected R15-3 cells. In the control S9-13 cells, the expression of HLA-1 was induced after IFN-α treatment.

### Efficient clearance of HCV replication from R15-3 cell line expressing IRF9-Stat fusion proteins

**Figure 6:**
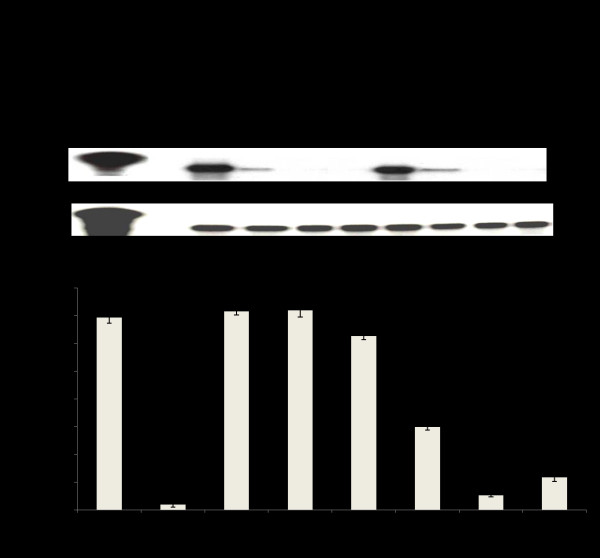
**Ribonuclease protection assay and Real-time RT-PCR of sub-genomic HCV 1b RNA**. **(A) **Intracellular expression of IRF9-S1C and IRF9-S2C or both in R15-3 cells inhibits HCV negative strand RNA detection by RPA. R15-3 cells were transfected treated (-) and (+) IFN-α (1,000 IU/ml), and total RNA was isolated at 72 hours post-transfection. The upper panel shows the RPA results of HCV negative strand detection. The bottom panel shows GAPDH mRNA levels by RPA. **(B) **Real-time RT-PCR was performed to quantify HCV RNA in R15-3 transfected cells. Cellular RNA was isolated at 72 hours, retrotranscribed, and assayed by real time PCR. S9-13 and R15-3 cells (+) and (-) IFN-α were used as (+) and (-) controls, respectively. Error bars represent SD. Asterisk (*) indicates transfected R15-3 cells with significantly lower HCV RNA relative to untransfected R15-3 cells treated with IFN. Significance was considered at p-values < 0.05.

The ability of the different IRF9-Stat fusion plasmids to inhibit viral replication was examined using stable replicon cell lines containing either HCV genotype 1b or HCV 2a sub-genomic RNA. RPA detection of the HCV negative strand RNA replication intermediate was used to assess viral replication in IRF9-Stat fusion transfected R15-3 cells. The RPA results demonstrate that intracellular expression of IRF9 alone had no effect upon HCV negative strand RNA levels, whereas all of the IRF9-Stat fusion constructs showed antiviral activity in an IFN-α independent manner (Fig. 6A). Transfection with IRF9-S1C reduced HCV negative sense RNA strand levels, and IRF9-S2C alone or in combination with IRF9-S1C inhibited HCV replication below the limit of detection of the RPA assay. The HCV negative strand RNA RPA results were verified by quantifying HCV positive strand RNA by real-time RT-PCR. The results in Fig. 6B demonstrate that R15-3 cells transfected with IRF9-S1C, IRF9-S2C, or the combination experienced a significant reduction (p < .05) in log copies of HCV RNA per μg of cellular RNA compared to R15-3 cells plus IFN-α (1,000 IU/ml). IRF9 alone transfected R15-3 cells and showed a modest reduction of HCV RNA. S9-13 cells with and without IFN-α (1,000 IU/ml) were used as a positive control.

**Figure 7:**
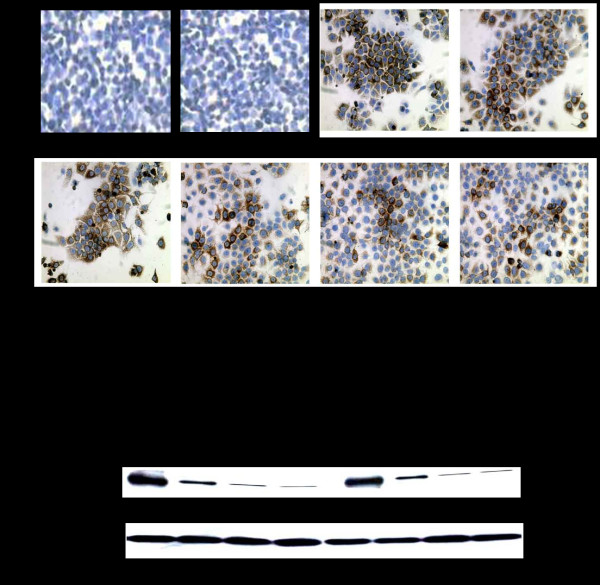
**Immunostaining of HCV NS3 and Western blot of HCV NS5B proteins**. **(A) **The antiviral activity of the IRF9-Stat fusion constructs in R15-3 cells containing HCV 1b sub-genomic RNA HCV. At 48 hours post-transfection the cells were mounted onto a glass slide, stained for HCV NS3 protein and counterstained with hematoxylin. Panel i and ii: Huh-7 cells (-) and (+) IFN-α (1,000 IU/ml). Panel iii and iv: R15-3 cells (-) and (+) IFN-α. Panel v: IRF9 transfected R15-3 cells Panel vi: IRF9-S1C transfected R15-3 cells Panel vii: IRF9-S2C transfected R15-3 cells Panel viii: IRF9-S1C plus IRF9-S2C transfected R15-3 cells. **(B) **Upper panel: HCV NS5B expression levels in R15-3 transfected cells at 72 hours post-transfection. Lower panel: Shows β-actin protein expression levels using equal amounts of protein lysate.

**Figure 8:**
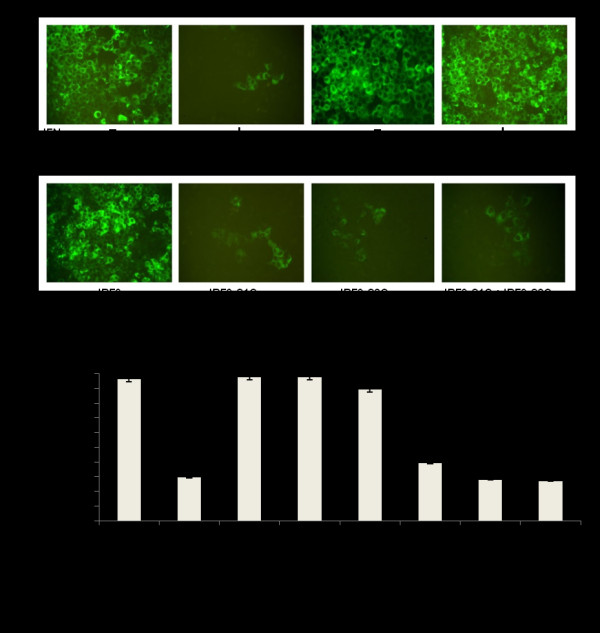
**The antiviral activity of the IRF9-Stat fusion constructs in an IFN resistant cell line containing HCV 2a sub-genomic RNA (R4-GFP)**. **(A) **GFP expression levels in R4-GFP cells transfected with IRF9, IRF9-S1C and IRF9-S2C at 72 hrs post-transfection. (**B) **Flow cytometric analysis of mean fluorescence intensity of R4-GFP transfected cells at 72 hrs. S3-GFP and R4-GFP cells treated with and without IFN-α were used as controls. The error bars represent the SEM from three experiments. The Student's t-test was used to compare R4-GFP transfected cells to R4-GFP cells plus IFN-α. P-values < 0.05 were considered significant.

**Figure 9:**
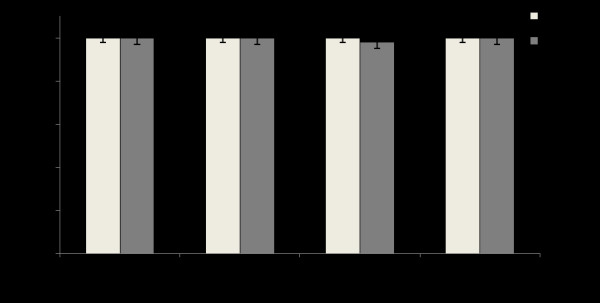
**MTT Assay of IRF9-Stat fusion constructs in resistant cell line**. R15-3 cells were transfected with IRF9 constructs and at 72 hours post-transfection cell viability was determined by the MTT assay. Each value represents experiments performed in triplicate. Error bars represent SEM.

Viral protein expression was assessed by immunocytochemical staining for HCV NS3 protein. The results presented in Fig. 7A suggest that the transfection of R15-3 cells using the individual constructs IRF9-S1C, IRF9-S2C or the combination reduced HCV NS3 protein expression in an IFN-α independent manner. The IRF9 alone-transfected R15-3 cells showed no reduction in viral protein levels while the IRF9-S1C, IRF9-S2C and the combination of both transfected R15-3 cells displayed a reduction of HCV NS3 expression (Fig. 7A). The addition of IFN-α had no effect on viral protein levels in each experimental group. Western blot analysis was then performed using an antibody against HCV NS5B to verify the results of the antiviral effect of the IRF9-Stat fusions. IRF9-S2C and IRF9-S1C plus IRF9-S2C reduced HCV NS5B protein levels in R15-3 cells (Fig. 7B). R15-3 cells transfected with IRF9 alone showed no reduction in HCV NS5B, while the IRF9-S1C transfected cells showed weak reduction in viral protein expression. All constructs exerted their antiviral activity in an IFN independent manner. The antiviral properties of the IRF9-Stat fusion constructs appear more robust at the 72 hour time point as compared to the previous immunocytochemical assessment of HCV NS3 at 48 hours. This increase in antiviral efficacy between these time points correlates with our experience with the antiviral activity of IFN-α in the S9-13 cell line. The antiviral effect of IRF9-Stat fusion protein expression in the HCV 2a-GFP resistant replicon cells (R4-GFP) was examined directly via fluorescence microscopy and then quantified by flow analysis (Fig. 8). It was determined that R4-GFP cells expressing IRF9 alone had no antiviral effects whereas all the remaining constructs had potential anti-HCV activity as they all inhibited HCV-GFP expression to various degrees. The strongest antiviral activity was observed in the IRF9-S2C transfected cells. The viability of the R15-3 cell line expressing different IRF9-Stat fusion constructs was then examined to exclude the possibility that the enhanced viral clearance was not due to the toxic effect of intracellular expression of the IRF9-Stat fusion proteins. The results presented in Fig. 9 suggest that no cellular toxicity was associated with the expression of the Stat fusion proteins in the R15-3 cells. Taken together, these results suggest that the intracellular expression of IRF9-S1C and IRF9-S2C leads to effective clearance of HCV RNA replication in an IFN-α resistant replicon cell line of both HCV1b and HCV2a genotype viruses.

**Figure 10:**
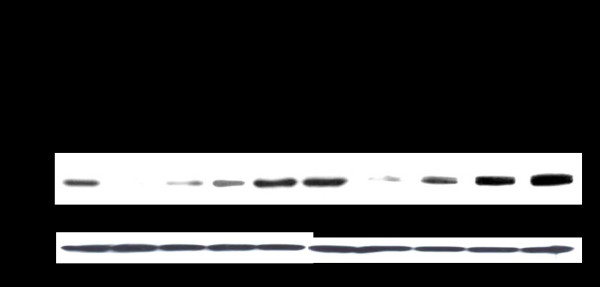
**Intracellular expression of IRF9-Stat fusion constructs induced eIF2α phosphorylation in the R15-3 cell line at 72 hours post-transfection**. Upper panel: Western blot of p-eIF2α in R15-3 cells transfected with different IRF9 plasmids in the absence and presence of IFN-α (1,000 IU/ml). Bottom Panel: β-actin protein expression levels.

Next, the mechanism of inhibition of viral RNA replication in cells expressing the IRF9-Stat fusions was examined. A principal mechanism that contributes to the antiviral state and which is activated upon ISRE promoter induction is the phosphorylation of eIF2α. Phosphorylation of eIF2α leads to translational inhibition within the cell thereby preventing viral replication. Therefore, we assessed the ability of each construct to induce p-eIF2α both in the presence and absence of IFN-α. The result shown in Fig. 10 suggests that IRF9 alone was unable to induce p-eIF2α, while IRF9-S1C caused low-level induction of p-eIF2α. Transfection of IRF9-S2C alone and IRF9-S1C plus IRF9-S2C caused high-level p-eIF2α induction. The addition of IFN-α did not significantly (p < .025) alter the expression patterns of p-eIF2α.

## Discussion

The molecular mechanisms of IFN-α resistance are unclear, however a number of studies have provided evidence that both viral and cellular factors are involved [18-23]. We provided evidence that replicon cells develop IFN-α resistance due to defective Jak-Stat signaling. Using long-term IFN-α treatment of replicon cells we have established IFN-α resistant sub-genomic HCV based stable replicon cell lines for HCV 1b and HCV 2a genotype viruses. Replication of HCV RNA in these stable cell lines remained resistant to IFN-α due to defects in Stat1 and Stat2 phosphorylation and the subsequent impairment of nuclear translocation of ISGF3. In this study, a potential therapeutic strategy against chronic HCV infection using a chimeric protein of IRF9 fused to the TAD of either the Stat1 or Stat2 molecule of the Jak-Stat signaling pathway was examined.

We showed that the IRF9 protein was expressed at intermediate levels in the R15-3 cells after transfection and efficiently localized to the nucleus, suggesting that the nuclear translocation of IRF9 protein in the R15-3 cells is not dependent upon IFN-α induced Jak-Stat signaling. Nuclear translocation of the Stat1-GFP and Stat2-GFP chimera proteins was efficient in the S9-13 cell line; however the nuclear localization of Stat1-GFP and Stat2-GFP did not occur in the R15-3 cells that possess defective Jak-Stat signaling. These results led us to examine whether the development of a chimera protein between the IRF9 and the TAD of either Stat1 or Stat2 protein could facilitate nuclear translocation and induce IFN antiviral gene expression. We showed here that intracellular expression of the IRF9-Stat1 and Stat2 fusion proteins in the R15-3 cell line activated the ISRE-luciferase promoter in a concentration dependent manner. Expression of the IRF9 protein alone did not activate the ISRE-luciferase promoter activity suggesting that IRF9 alone does not control the antiviral gene transcription induced by IFN-α. The results of this study also validate the previous finding that the fusion of the TAD of either Stat1 or Stat2 to IRF9 protein induced the ISRE promoter [14]. The activity of IRF9-S2C was stronger than IRF9-S1C. The recombinant IRF9-Stat fusion constructs also induced HLA-1 surface expression in R15-3 cells in an IFN-α independent manner. Based on these results we speculate that intracellular expression of IRF9-S2C or IRF9-S1C in HCV infected hepatocytes may induce HLA-1 surface expression and may thus increase cytotoxic T lymphocyte (CTL) mediated viral clearance. Interestingly we observed that the intracellular expression of IRF9-S2C alone or the combination with IRF9-S1C showed stronger activation of ISRE-luciferase promoter activation than IRF9-S1C alone, indicating that Stat2 expression contributes more towards the potent antiviral activity against HCV infected hepatocytes with defective Jak-Stat signaling. On the other hand, intracellular expression of either IRF9-S1C, IRF9-S2C or the combination of both show similar results in the mobilization of HLA-1 surface expression in R15-3 cells. These results suggest that intracellular expression Stat1 fusion could contribute more towards the immunomodulatory activity in HCV infected hepatocytes with defective Jak-Stat signaling.

The antiviral properties of the IRF9-Stat fusion proteins were also examined using stable IFN-resistant cell lines of HCV 1b and 2a genotype viruses. Using the R15-3 cell line containing sub-genomic HCV genotype 1b RNA, we showed that intracellular expression of IRF9-S1C and IRF9-S2C efficiently inhibited negative strand HCV RNA levels and viral NS3 protein expression in a replicon cell line containing defective Jak-Stat signaling. This approach was also tested using IFN-α resistant HCV 2a sub-genomic GFP replicon. We showed that transient transfection of IRF9-S1C and IRF9-S2C or both inhibited GFP expression in an IFN-α independent manner. These results suggest that this antiviral strategy may be effective against different HCV genotypes. To understand the mechanism of inhibition of HCV replication in the R15-3 cell line, the protein kinase R (PKR) mediated eIF-2α phosphorylation pathway was examined by western blot analysis. Our results suggest that the mechanism of inhibition of viral replication by intracellular IRF9-Stat fusion expression is similar to that described for IFN-α and involves PKR induced eIF-2α phosphorylation [24].

A critical determinant in the outcome of HCV infection is the host's ability to mount an effective HCV specific CD8+ T cell response. It was also previously demonstrated that a defective Jak-Stat system contributed to impaired HLA-1 surface expression [25]. It is therefore conceivable that the ineffective CD8+ responses of chronically infected HCV patients and IFN non-responders may in part be linked to the inability of infected hepatocytes to surface display the HLA-1: antigen complex. This impaired surface presentation may also contribute to the immune evasion observed in virally infected hepatocellular carcinoma cells. A substantial body of clinical evidence exists to support the role of dysregulated Jak-Stat signaling in IFN non-response in chronic HCV infection [26-29]. These clinical studies as well as our cell culture studies support the importance of Jak-Stat signaling in infected hepatocytes in viral persistence and response to IFN-α treatment. Using HCV cell culture models we now provide evidence that components of the Jak-Stat pathway can be engineered to circumvent defective signaling thereby inducing an antiviral state resulting in HCV eradication without IFN-α treatment. Based on these in vitro studies we propose that combination treatment using IRF9-S1C and IRF9-S2C recombinant proteins may provide a rationale for future development of an antiviral strategy to overcome IFN-α resistance by circumventing Jak-Stat cellular defects in chronically infected liver cells. We propose that liver targeted delivery of IRF9-Stat fusion protein can be used as a second line treatment in chronically infected hepatitis C patients with defective Jak-STAT signaling in an attempt to stimulate an antiviral response as well as increase HLA-1 expression in hepatocytes in an IFN-α independent manner.

## Competing interests

The authors declare that they have no competing interests.

## Authors' contributions

BP performed major biochemical experiments, participated in the design of the study and wrote the initial draft of the manuscript. SH, PKC, FG and XA did some biochemical experiments and participated in the design of the study. SD and RFG supervised, helped to design the study and finally wrote the manuscript. LAB share ideas and helped for manuscript preparation. All authors read and approved the final manuscript.
